# The People’s War Against Pandemic: protocol for a randomized control trial based on a virtual interactive training system intervention to improve the emergency preparedness of public for major emerging infectious diseases

**DOI:** 10.1186/s12889-023-15966-4

**Published:** 2023-06-01

**Authors:** Yue Luo, Wei Wei, Mei Li, Jianlan Ren, Yu Zheng, Yongli Huang, Yanhua Chen

**Affiliations:** 1 Department of Cardiology, West China Hospital, Sichuan University, Chengdu City, China; 2 West China School of Nursing, Sichuan University, Chengdu City, China; 3 School of Nursing, Southwest Medical University, Luzhou City, China; 4 Department of Gynecology, The First Affiliated Hospital of Chongqing Medical University, Chongqing City, China; 5 Department of Anesthesiology, The Affiliated Hospital of Southwest Medical University, Luzhou City, China; 6 Department of Rheumatism and Immunology, The Affiliated Hospital of Southwest Medical University, Luzhou City, China; 7 Outpatient Department, The Affiliated Hospital of Southwest Medical University, Luzhou City, China; 8 Department of Nursing, The Affiliated Hospital of Southwest Medical University, Luzhou City, China

**Keywords:** Major emerging infectious diseases, Emergency preparedness, Virtual interactive, Protocol

## Abstract

**Background:**

The frequent occurrence and increasing severity of major emerging infectious diseases (MEIDs) have posed considerable public health, economic and social issues worldwide. The emergency preparedness of public is inadequate to respond to and recover from MEIDs. Due to the limitation of time, space and resources, it is also difficult to carry out large-scale emergency preparedness training related to MEIDs. Then we developed a virtual interactive training system to improve emergency preparedness of public, including preparation of legal compliance, emergency knowledge, emergency capacity, economic estimation, material reserve and physical and mental health.

**Methods:**

A protocol for conducting a randomized controlled trail to evaluate the People’s War against Pandemic, a virtual interactive training system aimed to improve emergency preparedness of public for MEIDs. During the intervention, participants need to complete the storyline task at least once a day, watch at least one article and one video in the knowledge corner, and complete a retest of wrong choices in the intelligent evaluation module. The primary outcome is emergency preparedness of public for MEIDs. The secondary outcome is prevention and control knowledge of MEIDs.

**Discussion:**

The People’s War Against Pandemic may be an effective approach to provide public with a panoramic understanding of the response to MEIDs, so as to promote their comprehensive preparation and finally achieve effective response.

**Trial registration:**

This study was funded in 2021 and registered in the Chinese Clinical Trial Registry (registration number: ChiCTR2200060919) in June 2022. Recruitment and enrollment of participants began in July 2022.

## Background

In the past two decades, major emerging infectious diseases (MEIDs) including SARS, H7N9 avian influenza, Ebola fever, Middle East respiratory syndrome and Corona virus disease 2019 (COVID-19) have had considerable disastrous impact worldwide [1]. Particularly, COVID-19 has had an unprecedented impact on human lives and health, social stability and economic development [2]. Meanwhile, a series of global changes has increased the risk from MEIDs, making pandemics like COVID-19 likely to occur more frequently [3]. Public is the first responder to emergencies and is most likely to achieve self-rescue and mutual-rescue before and after emergencies [4]. To respond to and recover from the pandemics, it is necessary for public to make emergency preparedness.

In 2007, Nelson et al. [5] defined public health emergency preparedness as “the capability of the public health and health care systems, communities, and individuals to prevent, protect against, quickly respond to, and recover from health emergencies, particularly those whose scale, timing, or unpredictability threatens to overwhelm routine capabilities.” The object of emergency preparedness is not only the government and institutions, but also individuals at the public level. However, research on emergency preparedness of public in various countries shows that the public is usually underprepared [6–8], mainly reflected in lack of preparedness in emergency knowledge and skills. Due to the limitation of time, space and resources, it is also difficult to carry out large-scale emergency preparedness training related to MEIDs [9].

With the rapid development of mobile Internet and information technology, emergency education based on Internet plays an important role in public emergency response [10]. Among them, virtual interactive emergency training can break through the limitations of time, space and resources to provide the public with large-scale, homogeneous, low-cost and safe emergency preparedness training. In response to MEIDs, preventive medicine, nursing and emergency management department actively carry out virtual interactive training research [11, 12]. The training content of those studies is limited to the training objectives of professionals and lacks the real needs of emergency preparedness of public. In view of this, we constructed a training content framework for emergency preparedness of public for MEIDs including 20 knowledge, emotion and behavior tendency covering five aspects (cooperating with prevention and control work, improving emergency response ability, guaranteeing supplies and equipment, preparing economic resources, and maintaining physical and mental health), and developed a virtual interactive training system to improve emergency preparedness of public [13].

Below we describe a protocol of our randomized controlled trail to evaluate the effect of the virtual interactive training system intervention to improve emergency preparedness of public. The intervention, called The People’s War against Pandemic, is a 2-week virtual interactive training, designed to improve the ability of public to prevent, respond to and recover from MEIDs, including preparation of legal compliance, emergency knowledge, emergency capacity, economic estimation, material reserve and physical and mental health.

## Methods

### Study design

**Fig. 1:**
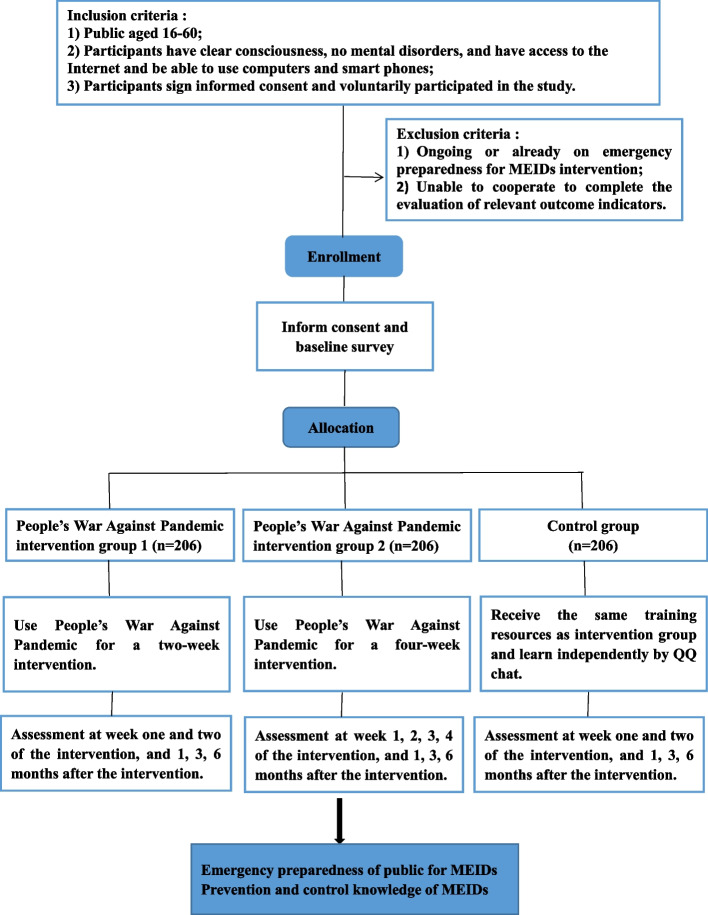
Study flowchart

The design type of this study is a randomized controlled trial. Study participants will be recruited from public volunteers and allocated to one of the three randomized groups after completing a baseline survey (Fig. 1) (The People’s War Against Pandemic intervention group 1, The People’s War Against Pandemic intervention group 2, Control group). Three follow-up visit assessments are required to complete for participants at four weeks later, three months and six months after the intervention. Our hypotheses are:

1. There is no statistical difference between The People’s War Against Pandemic intervention group 1 and The People’s War Against Pandemic intervention group 2 in prevention and control knowledge of MEIDs and emergency preparedness of MEIDs.
2. Comparing The People’s War Against Pandemic intervention group 1 and Control group, the former group will have better effect on improve prevention and control knowledge of MEIDs and emergency preparedness of MEIDs.
3. Comparing The People’s War Against Pandemic intervention group 2 and Control group, the former group will have better effect on improve prevention and control knowledge of MEIDs and emergency preparedness of MEIDs.

### Study participants

This study aims at improving the emergency preparedness of public for MEIDs. Public is the target population of this study. Inclusion criteria are 1) public aged 16-60; 2) Participants have clear consciousness, no mental disorders, and have access to the Internet and be able to use computers or smart phones; and 3) Participants sign informed consent and voluntarily participate in the study. Exclusion criteria include any of the following 1) Ongoing or already on emergency preparedness for MEIDs intervention; and 2) Unable to cooperate to complete the evaluation of relevant outcome indicators. Dropout criteria include any of the following 1) Participants lose contact and voluntarily withdrew from the study; and 2) Participants are unable to complete the study due to illness or accident.

### The People’s War Against Pandemic: overview

**Fig. 2:**
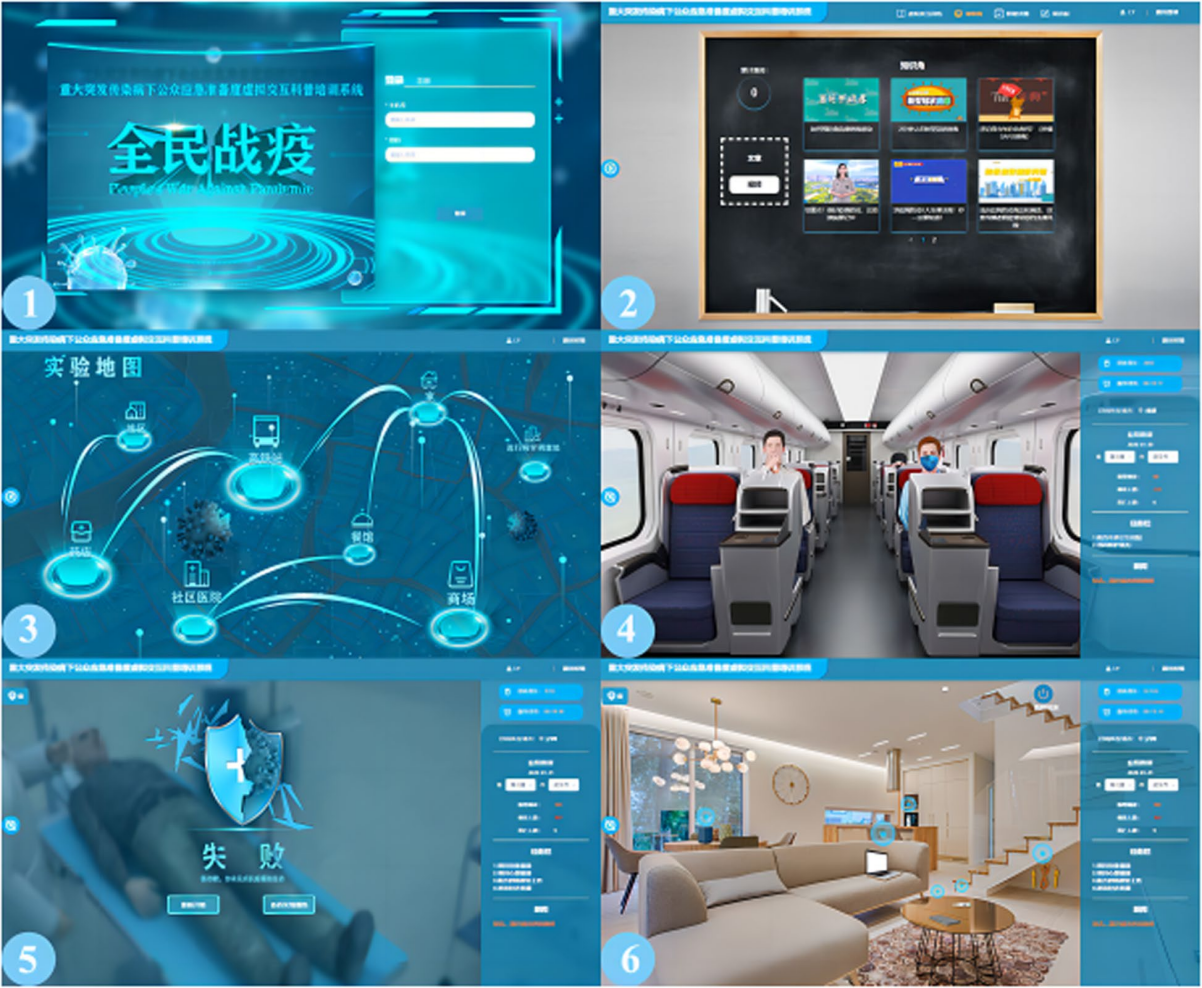
Interface of the system. (There are 6 interfaces in the picture. Interface 1 is the login interface; Interface 2 is the knowledge corner; Interface 3 is the map of different scenes; Interface 4 shows the scene of the high-speed railway carriage; Interface 5 is a warning interface for the virtual character infected with the disease after the training fails; Interface 6 shows the scene at home)

Under the guidance of multi-disciplinary theoretical framework (interactive narrative theory [14], situated learning theory [15], human-computer interaction theory [16]), we completed the scheme design and system development of virtual interactive training system for emergency preparedness of public for MEIDs. Participants can enter the system through computers, mobile phones, and tablets, and can log in for free after registration. The interface of the system is shown in the Fig. 2.

#### Training content

The training content index system was constructed based on the global national-level emergency preparedness index framework [17]. The training content index system includes five first-level indices (cooperating with prevention and control work, improving emergency response ability, guaranteeing supplies and equipment, preparing economic resources, and maintaining physical and mental health) and 20 s-level indices and their connotations including knowledge, emotion and behavioral tendency related to emergency preparedness for MEIDs.

#### Background storyline

The background storyline of the virtual interactive training system is the outbreak of a MEID in a certain place, and participants need to identify and analyze the harmfulness and controllability of the pandemic development according to the pandemic spread data. In each period of the pandemic spread, participants can act in each scene according to the storyline development and avoid infection with themselves and others through the correct selection in key interactive nodes. Participants can acquire knowledge and skills of prevention and control of MEIDs, adjust emergency emotion and change behavior tendency.

#### Module

1. Virtual interactive training module: This module is the core module of the system and the first module displayed after participants registration and login. Click start training to enter the background introduction interface, which introduces the purpose, requirements and steps of the training. Then enter the map interface, which is the display interface of each scene. Participants select the scene according to the prompt. Each scene has different tasks, which are set according to the training content index system. After the training task of one scene is completed, participants can choose to enter the next scene. During the training, each interactive interface will display the participants' current score (full score is 100), training time, current city, pandemic data and task bar. Participants make choices at interactive nodes based on the development and harmfulness of the pandemic. Every choice made at the interactive node is related to the success or failure of the training. The interactive interface of successful participants will prompt “you passed the training”, and those who have not passed will prompt “you failed”, and corresponding pictures will appear to show a series of disasters, including direct disasters (sick with the virus, pandemics spread globally) and indirect disasters (secondary disasters such as social, economic and cultural disasters), as a warning.
2. Knowledge corner module: This module contains knowledge and skills of MEIDs (including source of infectious, route of transmission, susceptible population, clinical manifestation, prevention and control measures), laws and regulations on the prevention and control of public health emergency, secondary disasters.
3. Intelligent evaluation module: This module records the behaviors and choices of participants in the training process and gives intelligent scores. Taking the training content indices as specific scoring points, the system judges each behavior and choice of the participants, scores the correct behavior or choice, and does not score the wrong one, and finally forms a training report. The training report shows a radar chart of training score, time, number of training sessions, and each behavior and choice. Wrong choices in the training process can be re-tested to achieve the purpose of consolidating knowledge.
4. Forum community module: Participants can leave insights, gains and experiences, system bugs, opinions and suggestions in this module.
5. User management module: This module integrates the task release, data collection and integration, account management and score management, which can provide real-time and objective data for the prevention and control of MEIDs.

### Intervention design: overview

#### The People’s War Against Pandemic intervention group 1

Participants are asked to use The People’s War Against Pandemic for a two-week intervention. Participants need to complete the storyline task at least once a day, watch at least one article and one video in the knowledge corner, and complete a retest of wrong choices in the intelligent evaluation module. Researchers check the completion of participants through the user management module, and those who fail to complete will be reminded by sending text messages or phone. The effect of the intervention will be evaluated by using the same questionnaire as applied in the baseline survey at week one and two of intervention. Before the training, participants will be informed that they will be rewarded with 50 RMB after completing daily tasks and questionnaire.

#### The People’s War Against Pandemic intervention group 2

Participants are asked to use The People’s War Against Pandemic for a four-week intervention. Other requirements are the same as those of intervention group 1. The effect of the intervention will be evaluated at week 1, 2, 3, 4 of intervention. The purpose of setting this group is to explore the optimal duration of the training, so that the participants will neither fail to achieve the training effect due to too short time nor get bored due to too long time.

#### Control group

We established a QQ chat group and invited participants into this group. The same training resources as emergency preparedness training content index system and the knowledge corner are pushed to participants, and participants are required to study independently and complete online check-in for 2 weeks. Participants will be rewarded with 50 RMB after completing daily tasks and questionnaire, just like the intervention group. But this group does not have elements of storylines, virtual scenes, interactions and scoring.

### Recruitment, randomization, and allocation concealment

#### Recruitment

Participants will be recruited through both online and on-site recruitment. The sample size calculation is based on the primary outcome: emergency preparedness of public for MEIDs. The sample size was performed using the formula developed by Chow [18], and the formula is as follows:

$$\begin{array}{c}\mathrm{n}=2{\left(\sigma \frac{{z}_{1-\alpha /\left(2T\right)}+{z}_{1-\beta }}{{\mu }_{A}-{\mu }_{B}}\right)}^{2}\\ 1-\beta =\varphi \left(z-{z}_{1-\alpha /\left(2T\right)}\right)+{\varphi }\left(-z-{z}_{1-\alpha /\left(2T\right)}\right), z=\frac{{\mu }_{A}-{\mu }_{B}}{\sigma \sqrt{\frac{2}{n}} }\end{array}$$

The significance level of the test is α = 0.05, the test power is 1-β = 0.8, standard deviation is = 26.31, the number of comparisons to be made is T = 3, is the standard Normal distribution function. Calculator software (Power and Sample Size) was used to calculate the initial sample size N1 = 171. Considering the attrition rate of 20%, the sample size of each group was increased to 206, and the total sample size of the three groups was 618.

The study will be conducted in Luzhou City, Sichuan Province, China. Participants were recruited from the community and signed informed consent forms if they agreed to participate in the study and complete follow-up measurements. After completing the study, each participant will receive a reward of 50 RMB in cash.

#### Randomization and allocation concealment

To reduce selection bias, randomization and allocation concealment are essential. To achieve randomization, there are the following steps: 1) Sequence numbers are drawn up for 618 participants; 2) 618 random numbers are randomly generated by computer; 3) 618 random numbers are finally distributed to 618 participants. Opaque envelopes are used to allocation concealment. In each of the 618 envelopes, each will contain a note with the words “The People’s War Against Pandemic intervention group 1” or “The People’s War Against Pandemic intervention group 2” or “Control group”(206 envelopes per group). Participants will randomly select an envelope in the order of random number and be assigned to the corresponding group according to the information in the envelope. The grouping information is saved by a third party. Unlinding is permissible when participants exit the research or statistical analysis is completed.

### Outcomes and data collection

**Fig. 3:**
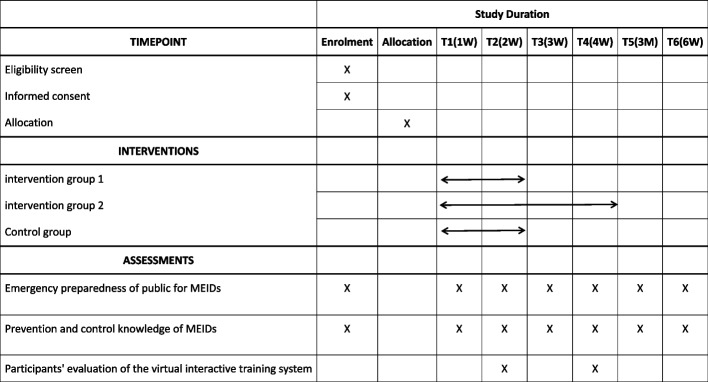
Study schedule of enrolment, interventions, and assessments

The primary outcome is emergency preparedness of public for MEIDs. The secondary outcome is prevention and control knowledge of MEIDs. The assessment of intervention group 1 will be conducted at baseline, week one and two of the intervention, and 1, 3, 6 months after the intervention. Assessment of the intervention group 2 will be conducted at baseline, week 1, 2, 3, 4 of intervention, and 1, 3, 6 months after the intervention. Assessment of the control group will be conducted at baseline, week one and two of the intervention, and 1, 3, 6 months after the intervention. In addition, participants' evaluation of the virtual interactive training system will be assessed at the completion of the intervention. Study schedule of enrolment, interventions, and assessments is shown in the Fig. 3.

Emergency preparedness of public for MEIDs will be assessed with a questionnaire. The items of the questionnaire are designed according to the training content index system (five first-level indices, 20 s-level indices and their connotations). Six experts in infectious diseases, public health and psychology were invited to evaluate the content validity of the questionnaire, and the item-level content validity index (I-CVI) and the scale-level content validity index (S-CVI) were both 1.000. There are 53 items in total. Likert five-level scale was used for each item, with 1 to 5 points representing “strongly disagree”, “disagree”, “uncertain”, “agree” and “strongly agree”, respectively.

Prevention and control knowledge of MEIDs will be assessed with a questionnaire including basic knowledge of prevention and control of MEIDs (source of infectious, route of transmission, susceptible population, clinical manifestation, prevention and control measures), laws and regulations on the prevention and control of public health emergency, secondary disasters. After the expert consultation, the I-CVI and the S-CVI were both 1.000. There are 17 items in total, 1 point for a correct answer. The higher score means the more knowledge is learned by the participants.

Participants' evaluation of the virtual interactive training system will be assessed with a questionnaire including interactivity, usability, immersion and education of the system. After the expert consultation, the I-CVI and the S-CVI were both 1.000. There are 17 items in the questionnaire, including four parts: interactivity (four items), usability (four items), immersion (four items) and education (five items). Likert five-level scale was used for each item, with 1 to 5 points representing “strongly disagree”, “disagree”, “uncertain”, “agree” and “strongly agree”, respectively.

### Data management and statistical analysis

Data will be collected, recorded and managed by the Survey Star, an online questionnaire platform (www.wjx.cn, Changsha Ranxing Information Technology Co., Ltd in China) to ensures the safety, integrity and consistency of the data. Researchers will download the data from Survey Star and save it in text format to a password-protected server. SPSS 21.0 analysis software was used for data analysis by the third party who did not participate in the intervention.

Data will be collected at baseline and descriptive statistics will be used to show the characteristics of each arm at baseline. The outcome indicator prevention and control knowledge of MEIDs is a binary variable, so the chi-square test will be used to compare among the three arms at the intervention completion, 1, 3, and 6 months after the intervention. Analysis of Variance (ANOVA) will be used to find out whether there are differences among the three arms in the outcome indicator-emergency preparedness of public for MEIDs. Repeated Measures ANOVA will be used to measure weather there are differences in emergency preparedness of public for MEIDs score at 1, 3, and 6 months after intervention. A Bonferroni correction will be used for multiple testing. Intention-to-treat analysis will be used to account for the influence of attrition. The difference was statistically significant when *P* ≤ 0. 05.

### Trial registration

This study was funded in 2021 and registered in the Chinese Clinical Trial Registry (registration number: ChiCTR2200060919) in June 2022. Recruitment and enrollment of participants began in July 2022.

### Dissemination policy

The results will be disseminated in the form of a paper or research report. After testing and review, The People’s War Against Pandemic will be provided to public free of charge online. The original data will be uploaded to the China Clinical Trial Registry, where the original data can be viewed by searching the study registration number.

## Discussion

WHO encourages individuals to be educated in the necessary knowledge and skills related to emergency preparedness, especially in areas where emergency preparedness is weak [19]. In the pandemic of a MEID, the emergency preparedness of public was not enough to support the public's response and recovery [20]. On the one hand, the public usually receives publicity on emergency knowledge, emergency skills and preventive measures of MEIDs in daily life [21], and lacks emergency preparedness education on social cooperation, legal compliance, economic estimation, material reserves and physical and mental health. On the other hand, the evaluation indicators of emergency preparedness for MEIDs in China pay more attention to the preparation of knowledge and ability but ignore the preparation of emotion and behavior tendency [22]. This study provides the public with a panoramic understanding of the response to MEIDs, to promote their comprehensive preparation and finally achieve an effective response.

To meet the educational need of emergency preparedness of MEIDs for public, a virtual interactive training system The People’s War Against Pandemic was developed. As mentioned above, The People’s War Against Pandemic is a theory-based virtual interactive training system that can provide emergency knowledge, emergency skills, preventive measures, emergency emotion and behavior tendency to public to response and recover from MEIDs. Whether public are interested in The People’s War Against Pandemic and insist on using it is the key to improve the emergency preparedness of public. If the virtual interactive training system does not appeal to public, it will not be popularized among them, nor have educational significance. To promote the use of the virtual interactive training system by public, some elements are used to design the system, including storylines, virtual scenes, and interactions and scoring. This research realizes the interaction between human and computer by publishing tasks, independent selection and timely feedback. Compared with other training systems [23]. The People’s War Against Pandemic supports users to choose by themselves at the interactive node to determine the direction of training, which can mobilize users' subjective initiative and stimulate their learning motivation.

Three arms were designed in this research, including two intervention arms and one control arms. Participants in the two intervention groups will receive The People’s War Against Pandemic to complete the storyline task at least once a day, read at least one article and watch one video in the knowledge corner, and complete a retest of wrong choices in the intelligent evaluation module. The difference between the two groups is the intervention time. Due to the interactivity of virtual interactive training system, participants may have different outcomes in different training. To explore the optimal duration to finish all the outcomes, we designed two-week and four-week groups, so that the participants will neither fail to achieve the training effect due to too short time nor get bored due to too long time. By comparing the intervention groups with the control group, it can be found whether The People’s War Against Pandemic is better than autonomous learning in improving emergency preparedness of MEIDs for public. By comparing the two intervention groups, we can find out the optimal duration to improve emergency preparedness of MEIDs for public.

### Limitations

As with any research design, the trial also has potential limitations. In the follow-up observation, the time may be too long, to loss of participants. We can reduce the dropout rate by strengthening the connection with participants or giving appropriate rewards. In addition, The People’s War Against Pandemic has a scoring element, and participants may be addicted to pursuing high scores. To address this problem, we can limit the time of training every day.

## Data Availability

Not applicable.

## Abbreviations

MEIDs: Major emerging infectious diseases
COVID-19: Corona virus disease 2019
I-CVI: Item-level content validity index
S-CVI: Scale-level content validity index
ANOVA: Analysis of Variance
